# Inoculation insensitive promoters for cell type enriched gene expression in legume roots and nodules

**DOI:** 10.1186/s13007-016-0105-y

**Published:** 2016-01-22

**Authors:** Srdjan Gavrilovic, Zhe Yan, Anna M. Jurkiewicz, Jens Stougaard, Katharina Markmann

**Affiliations:** Department of Molecular Biology and Genetics, Centre for Carbohydrate Recognition and Signalling (CARB), Aarhus University, Gustav Wieds Vej 10, 8000 Aarhus, Denmark

**Keywords:** Cell type specific expression, Cell type enriched expression, Cell type specific promoter activity, Cell type enriched promoter activity, Nodulation symbiosis, *Lotus* root structure, *Lotus* nodule structure, *Lotus japonicus*, Legume

## Abstract

**Background:**

Establishment and maintenance of mutualistic plant–microbial interactions in the rhizosphere and within plant roots involve several root cell types. The processes of host–microbe recognition and infection require complex signal exchange and activation of downstream responses. These molecular events coordinate host responses across root cell layers during microbe invasion, ultimately triggering changes of root cell fates. The progression of legume root interactions with rhizobial bacteria has been addressed in numerous studies. However, tools to globally resolve the succession of molecular events in the host root at the cell type level have been lacking. To this end, we aimed to identify promoters exhibiting cell type enriched expression in roots of the model legume *Lotus japonicus*, as no comprehensive set of such promoters usable in legume roots is available to date.

**Results:**

Here, we use promoter:*GUS* fusions to characterize promoters stemming from Arabidopsis, tomato (*Lycopersicon esculentum*) or *L.japonicus* with respect to their expression in major cell types of the *L.japonicus* root differentiation zone, which shows molecular and morphological responses to symbiotic bacteria and fungi. Out of 24 tested promoters, 11 showed cell type enriched activity in *L.japonicus* roots. Covered cell types or cell type combinations are epidermis (1), epidermis and cortex (2), cortex (1), endodermis and pericycle (2), pericycle and phloem (4), or xylem (1). Activity of these promoters in the respective cell types was stable during early stages of infection of transgenic roots with the rhizobial symbiont of *L.japonicus*, *Mesorhizobium loti.* For a subset of five promoters, expression stability was further demonstrated in whole plant transgenics as well as in active nodules.

**Conclusions:**

11 promoters from Arabidopsis (10) or tomato (1) with enriched activity in major *L.japonicus* root and nodule cell types have been identified. Root expression patterns are independent of infection with rhizobial bacteria, providing a stable read-out in the root section responsive to symbiotic bacteria. Promoters are available as cloning vectors. We expect these tools to help provide a new dimension to our understanding of signaling circuits and transcript dynamics in symbiotic interactions of legumes with microbial symbionts.

**Electronic supplementary material:**

The online version of this article (doi:10.1186/s13007-016-0105-y) contains supplementary material, which is available to authorized users.

## Background

**Fig. 1:**
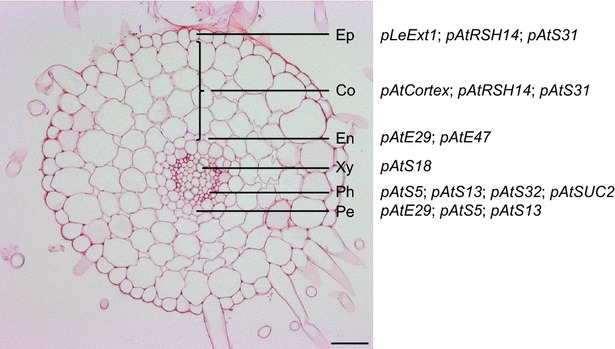
Cross-section of *L.* *japonicus* root (differentiation zone). *Co* cortex, *En* endodermis, *Ep* epidermis, *Pe* pericycle, *Ph* phloem, *Xy* xylem. Names of promoters are indicated in association with the cell type(s) where promoter-GUS activity was detected in *L. japonicus*. Promoters are underlined to indicate the cell type of primary expression. *Scale bar* 50 µm

Plant roots are dynamic structures involved in diverse developmental and physiological processes [1]. Apart from their roles in water and nutrient homeostasis, roots perceive biotic and abiotic environmental factors from the rhizosphere, and mediate appropriate responses [2–4]. Roots grow from an apical meristem at the tip and can be divided into developmental zones along their longitudinal axis [5]. The root apical meristem shows high rates of cell proliferation and is followed by the elongation zone, where meristematic activity ceases while cell elongation continues to increase cell length. Further distal from the tip, in the differentiation zone, rapid cell elongation has terminated and cells begin to acquire distinct cellular identities along the radial axis of the root [4, 5]. Major cell types are the peripheral epidermis, often including both root hair-developing trichoblasts as well as atrichoblasts, the cortex including the endodermis as its innermost cell layer, the pericycle, and the central vasculature with phloem and xylem elements for rootward and shootward long-distance transport, respectively [6] (Fig. 1). These distinct cell types differentially contribute to the functionality of the organ and plant as a whole [1, 4]. Only root cap and epidermis are in direct contact with the rhizosphere, but plant responses to stimuli also involve internal cell types such as cortex, endodermis, pericycle and vasculature [3].

The generation and maintenance of cellular identities in the root are determined by cell type dependent patterns of transcriptional activity [7–10], complemented by post-transcriptional control such as miRNA-mediated regulation of mRNA stability and translation [6]. Lee et al. [6] compared the cell type distribution of mRNAs of 44 Arabidopsis transcription factors to the respective promoter activity patterns in roots. The results suggested that for 80 % of investigated genes, about three kilobases of noncoding sequence upstream of the translation start site were sufficient to reproduce mRNA abundance patterns observed by hybridization-based gene-chip analysis [6, 10]. This observation implies that in the remaining 20 %, regulatory elements other than the five promoter regions contributed to mRNA patterning [6]. Beyond differential patterns of transcriptional and/or translational activity, the coordination of developmental processes requires intercellular communication and signal transduction between root cell types, in which diverse molecules including hormones [11–14], peptides [15–17] and regulatory RNAs [17, 18] are involved.

Many plants engage in synergistic interactions with bacterial or fungal microorganisms in their root systems. Legumes, known for their ability to form nitrogen-fixing root symbiosis, are responsive to compatible rhizobial bacteria within a section of the differentiation zone of the root referred to as the susceptible zone, that is transcriptionally distinct from other parts of the root [19]. The legume-rhizobia interaction involves the initiation of nitrogen-fixing nodule organs by the host plant, often preceded by deformation and curling of root hairs during epidermal infection [20]. In the model legume *Lotus japonicus*, *Mesorhizobium loti* bacteria usually invade the root through tube-like structures, infection threads, which initiate within root hair curls [21]. Infection progresses into and through cortical cell layers, and cortical cytokinin perception and signaling are required for reactivation of cell division [22, 23]. Within days of infection initiation, nodule primordia form in the root cortex. As their development progresses, bacteria invade the central primordial tissue through ramifying infection threads. Membrane-bound units of one or more bacteria are eventually released from infection thread tips into nodule cells, where they develop into nitrogen-fixing symbiosomes [24].

To dissect gene expression patterns in legume root interactions with microbial symbionts, previous studies have relied on laser microdissection of defined cell pools or tissue fragments to investigate mRNA or protein populations therein [25–30]. Though specific, such approaches are elaborate, and allow for the processing of limited sample numbers only. In addition, they rely on the availability of expensive equipment.

Here, we present a set of promoters showing cell type enriched expression in one or more cell types including epidermis, cortex, endodermis, pericycle, phloem poles and xylem elements of *L.japonicus* roots. These can be used for targeted isolation of cells for global transcriptome, proteome or metabolite analysis, as well as for directed expression of genes of interest in particular cell types. Activity patterns of the promoters predominantly originating from Arabidopsis or tomato (Table 1) have been tested using *GUS* reporter gene fusions in transgenic roots of composite plants [31]. Infection with *M.loti* demonstrated that expression patterns were stable upon rhizobial infection at the early, pre-nodulation stage of 3 days post infection. A subset of promoter:*GUS* constructs was used to generate whole-plant transformants, and expression patterns in transgenic lines were confirmed to resemble those in transgenic roots of composite plants. These stable lines were further used to analyze expression activity of the respective promoters in young and mature nodules harvested at 14 days post infection. The collection of promoters established and tested here is a valuable toolbox for directing expression in particular root and nodule cell types or cell type combinations, and for global analyses of how particular cell types contribute to symbiosis and organ development in *L.japonicus* and related legume species.

**Table 1:** Promoters tested for cell type enriched expression in *L. japonicus* roots

Predominant expression in *L. japonicus* roots	Promoter name	Promoter fragment used (bp)	Sequence origin (species)	Locus/accession	Gene product	References
Epidermis (trichoblast/atrichoblast)	*pLeExt1* ^a,b ^	1122	Tomato	NM_001247899	Extensin/xyloglucanv endotransglycosylase	Bucher et al. [32], Mirabella et al. [33]
Epidermis and cortex	*pAtS31*	3041	Arabidopsis	At5g19790	ERF/AP2 TF	Lee et al. [6]
	*pAtRHS14*	1249	Arabidopsis	At4g22080	Pectin lyase	Won et al. [35]
Cortex	*pAtCortex* ^a^	1666	Arabidopsis	At1g09750	Aspartyl protease	Lee et al. [6], Dinneney et al. [4]
Endodermis and pericycle	*pAtE29* ^a^	2724	Arabidopsis	At4g05170	bHLH TF	Lee et al. [6]
	*pAtE47*	3296	Arabidopsis	At2g37950	C3H TF	Lee et al. [6]
Pericycle and phloem	*pAtS5*	2202	Arabidopsis	At5g24800	bZIP TF	Lee et al. [6]
Phloem and pericycle	*pAtS32*	3000	Arabidopsis	At2g18380	C2C2-Gata TF	Lee et al. [6]
	*pAtS13* ^a^	4018	Arabidopsis	At1g07640	Dof TF	Lee et al. [6]
	*pAtSUC2* ^c^	942	Arabidopsis	At1g22710	Sucrose-proton symporter	Stadler et al. [37]
Proto- and metaxylem	*pAtS18* ^a^	3010	Arabidopsis	At5g12870	MYB TF	Lee et al. [6]
Unspecific	*pLjPDC1*	2987	*L. japonicus*	Lj1g2372300	Pyruvate decarboxylase	This manuscript
	*pAtE30*	3004	Arabidopsis	At4g21340	bHLH TF	Lee et al. [6]
	*pAtE31*	1484	Arabidopsis	At4g28890	C3H TF	Lee et al. [6]
	*pAtE49* ^d^	2414	Arabidopsis	At3g05150	C2H2 TF	Lee et al. [6]
Unstable (phloem)	*pAtS8* ^e^	3082	Arabidopsis	At5g60200	C2C2-Dof TF	Lee et al. [6]
Unstable (proto- and metaxylem)	*pAtS20* ^f^	3002	Arabidopsis	At1g71930	NAC TF	Lee et al. [6]

Where promoter activity was detected in more than one cell type, the cell type of primary expression is underlined. *Arabidopsis*, *A. thaliana*; tomato, *Lycopersicon esculentum*; TF, transcription factor

^a^Available in transgenic lines expressing *GUS*

^b^For *pLeExt1,* expression is enhanced in trichoblasts as compared to atrichoblasts

^c^Expression of *pAtSUC2* was exclusively seen in roots containing chloroplasts due to exposure to light

^d^ *pAtE49* shows unstable expression across most root cell types

^e^Expression of *pAtS8* is primarily associated with phloem poles, but occasional unspecific expression across root cell types is observed

^f^ *pAtS20* showed rare xylem associated expression. Where promoter activity was detected in more than one cell type, the cell type of primary expression intensity is underlined

## Results and discussion

### Isolation of promoters with cell type enriched expression patterns in *L.japonicus* roots

To identify promoters showing activity in one or more defined cell types in *L.japonicus* roots (Fig. 1), we assembled candidates based on their published expression properties in other plant species. These were then tested for their potential to drive expression of the *E.coliβ*-*glucuronidase* (*GUS*) reporter gene in *L.japonicus* roots in a cell type enriched manner. A total of 17 out of 24 tested promoters induced GUS activity in *L.japonicus* transgenic roots on composite plants (Table 1). To determine promoter activity patterns, entire transgenic roots were analyzed longitudinally. Root zones responsive to rhizobial infection, identified by the presence of immature, developing root hairs (differentiation zone), were cross-sectioned to visualize expression patterns in inner root cell types.

#### Epidermis enriched activity

One of the tested promoters, *pLeExt1* [32, 33], showed strongly enriched activity in epidermal cells. This promoter is the upstream regulatory sequence of an extensin/xyloglucan endotransglycosylase in tomato [33]. Transgenic roots transformed with the *pLeExt1:GUS* reporter construct (Additional file 1: Fig. S1.1a–f) showed GUS staining in young epidermal cells just prior to or coinciding with root hair emergence (Additional file 1: Fig. S1.1a and d). Epidermal activity was equally seen in the responsive zone containing developing root hairs (Additional file 1: Fig. S1.1b, c, e and f), as well as in older root zones containing mature hairs. In contrast to the previously documented trichoblast specific activity pattern in tomato roots, *pLeExt1:GUS* activity was slightly enhanced in *L.japonicus* trichoblasts as compared to atrichoblasts, but present in both.

Root patches devoid of traceable activity were present across developmental zones. In tendency, root sections with high root hair density showed enhanced expression. In line with this, *pLeExt1:GUS* activity in young epidermal cells depended on the pattern of root hair emergence on a particular root tip. Where root hairs developed in the vicinity of the root tip, epidermal cells tended to show GUS activity from an early and overall similar age resulting in a distinct ring of blue encircling the root tip (Additional file 1: Fig. S2), or expression activity developed more gradually (Additional file 1: Fig. S1.1a and d). Semithin cross sections of the responsive zone (7–8 µm) revealed some unspecific staining in other root cell types, mainly the hypodermal layer of the cortex (Additional file 1: Fig. S1.1c and f). Promoter regions of two *L.japonicus* homologs of the Arabidopsis expansin gene *AtExpa7*, *LjExpa7* and *LjExpa8,* have previously been shown to have strongly enriched activity in epidermal trichoblasts in *L.japonicus* roots [34]. Depending on the employed assortment of promoter fragments and root hair specific *cis* elements therein, activity was almost exclusive to root epidermal cells, or accompanied by varying levels of expression in the outer root cortex and central vasculature [34].

#### Epidermis and cortex enriched activity

*pAtS31* [6] (Additional file 1: Fig. S1.1g-l) and *pAtRHS14* [35] (Additional file 1: Fig. S1.1m-r) are promoter sequences of a pectin lyase and an ERF/AP2 (Ethylene Responsive Factor/APETALA2) transcription factor, respectively, in Arabidopsis. When coupled to a *GUS* reporter gene, both showed an overall similar activity pattern to *pLeExt1:GUS*, but apart from epidermal expression, that in cortical cells was more pronounced than observed in *pLeExt1:GUS* expressing roots (Additional file 1: Fig. S1.1c, f, i, l, o and r). Epidermal GUS signal was detected in both trichoblasts and atrichoblasts in roots expressing either promoter:*GUS* construct. In addition, *pAtS31:GUS* showed activity in one or more outer cortical cell layers proximal to the epidermis in all examined samples (Additional file 1: Fig. S1.1i and l), whereas *pAtRHS14* activity was consistently detected in cells across the root cortex, but not in the central vascular cylinder (Additional file 1: Fig. S1.1o and r). In roots expressing either construct, patches with pronounced promoter activity alternated with patches where activity was absent.

#### Cortex enriched activity

*pAtCortex* [4, 6], driving expression of an aspartyl protease gene originally isolated from Arabidopsis and named after its cortical expression in this species, had cortex enriched activity also in *L. japonicus* roots when coupled to a *GUS* reporter (Table 1, Additional file 1: Fig. S1.2a-f). Activity predominated in the inner three to four cortical cell layers peripheral of the endodermis, but included, at a weaker level, both the innermost endodermal and outermost hypodermal layers of the cortex. Some expression was present in the epidermis and vasculature as well as pericycle (Additional file 1: Fig. S1.2c and f), but GUS levels were much lower in these cell types than in the inner root cortex. Predominance of activity in the root cortex was particularly evident from thicker (60–80 µm) cross sections of the differentiation zone (Additional file 2: Fig. S3).

An additional promoter with documented cortex enriched (>90 %) expression in Arabidopsis, *pAtE49* [6], showed unspecific and unstable expression in *L.japonicus* roots (Table 1) and was thus not considered further.

#### Endodermis and pericycle enriched activity

*pAtE29* and *pAtE47* both show endodermal expression in the differentiation zone of Arabidopsis roots [6] and *GUS* marker gene fusions of these promoters had comparable endodermis enriched activity in *L.japonicus* (Additional file 1: Fig. S1.2g-r). GUS signal was also detected in the pericycle and, more weakly, in adjacent cells of the inner cortex of the differentiation zone (Additional file 1: Fig. S1.2i, l, o, r). In roots carrying the *pAtE29*:*GUS* construct, low signal levels were also present in more peripheral cortical layers as well as the epidermis, suggesting weak activity of the promoter in these cell types. In contrast, GUS signal was seen in endodermis, pericycle and, weakly, inner cortical cells only in *pAtE47*:*GUS* expressing roots. While an average of 62 % of roots containing the *pAtE29*:*GUS* construct showed *GUS* expression, activity was detected in an average of 43 % of roots transformed with *pAtE47*:*GUS* (Additional file 2: Table S2). This suggests less consistent activity of the latter promoter in *L.* *japonicus* roots, possibly due to a more pronounced dependence of its activation on the genomic location of the transgene.

#### Pericycle and phloem enriched activity

The promoter regions *pAtS5*, *pAtS32* and *pAtS13* [6] of three Arabidopsis transcription factors belonging to bZIP (Basic Leucine Zipper Domain), DOF (DNA-binding One Zinc Finger) and C2C2-GATA (containing either one or two zinc finger DNA-binding domains) families, respectively, showed pericycle and/or phloem enriched expression in *L.japonicus* roots. GUS signal was particularly pronounced in, but not limited to, the pericycle in *pAtS5*:*GUS* expressing roots (Additional file 1: Fig. S1.3a-f). Strong expression focused in pericycle cells opposite the phloem poles and incorporated cells of the latter. Less intense activity was apparent in *pAtS5*:*GUS* expressing pericycle cells opposing xylem poles, and occasional, weak GUS activity was present in inner cortical cells (Additional file 1: Fig. S1.3c,f). Expression of this promoter region in Arabidopsis roots similarly focused on the pericycle with about 62 % of the total observed intensity, whereas just above 20 % of detected expression intensity was phloem-associated [6]. A similar staining pattern, with more pronounced activity in the phloem cells but consistent, less intense GUS activity also in the associated pericycle, was observed in *pAtS32*:*GUS* expressing *L.japonicus* roots (Additional file 1: Fig. S1.3g-l). In Arabidopsis, more than 80 % of the detected *pAtS32* activity was phloem-associated, with very little expression present in the pericycle [6]. A similar activity pattern was detected in tomato [36].

The promoter region *pAtS13* was, in line with its expression pattern in Arabidopsis roots [6], primarily active in the phloem in *L.japonicus* roots, with limited amounts of GUS signal detected in phloem-associated pericycle cells of *pAtS13:GUS* expressing roots (Additional file 1: Fig. S1.3 m-r).

Similarly, a fourth promoter tested, *pAtSUC2* [37], induced GUS activity in phloem cells of *pAtSUC2:GUS* expressing *L.* *japonicus* roots as well as, to a lesser extent, in associated pericycle cells (Additional file 1: Fig. S4). However, expression activity was only apparent in roots that had been exposed to light and contained chloroplasts (Additional file 1: Fig. S4c-e), whereas GUS signal was entirely absent from *pAtSUC2*:*GUS* containing roots that had been shaded from light access and were photosynthetically inactive (Additional file 1: Fig. S2a-b). As root exposure to light potentially influences nodulation in both *L. japonicus* [38] and pea [39] we did not consider this promoter as a prime candidate. Our observations are consistent with the proposed function of *At*SUC2, a glucoside-proton symporter [40], in phloem loading [41].

#### Xylem enriched activity

The activity of *pAtS18*, regulating the expression of *At*MYB46, a member of the R2R3/MYB (containing an R2R3-type MYB DNA-binding domain) transcription factor family, was, as far as we could observe, limited to xylem elements within the central stele in *L.japonicus* roots (Additional file 1: Fig. S1.4a-f). Activity encompassed both proto- and metaxylem elements across the elongation and differentiation zones. This corresponds to the activity pattern observed in both Arabidopsis [6] and tomato [36]. Signal intensity was low and only detected in about 27 % of transgenic roots containing the *pAtS18*:*GUS* construct (Additional file 2: Table S1).

#### Activity patterns are stable during initial stages of rhizobial infection

For the investigation of cell type dependent responses during early stages of infection of *L.japonicus* roots with the rhizobial symbiont *M.loti*, candidate promoters with stable, infection-independent expression patterns are favorable, as they enable the targeting of similar cell pools across treatments. We therefore analyzed expression patterns in response to infection with *M.loti* at the early stage of 3 days post infection where existent cell types are comparable and nodule organogenesis has not yet commenced. All promoters showed stable activity patterns upon *M.loti* inoculation as compared to mock-treatment (Additional file 1: Fig. S1), suggesting their suitability for targeted expression studies or selective isolation of cell pools during early stages of rhizobial infection. Although the average percentage of transgenic roots with detectable GUS signal varied in inoculated compared to mock treated plants for some promoters (Additional file 2: Table S1), these differences were not significant due to overall high variation levels.

Some of the tested promoters have been shown in Arabidopsis to respond to exogenous stimuli including wounding or insect attack (*pAtS13*) [42], or to fungal (*pAtS18*) [43, 44] or viral (*pAtCortex*) [45] pathogen infection. Expression stability in response to other soil microbes such as arbuscular mycorrhizal fungi, or to abiotic challenges like nutrient levels, water- or temperature regimes will be interesting aspects of further investigation to broaden the applicability of this toolset.

#### Activity patterns are stable in transgenic plants

**Fig. 2:**
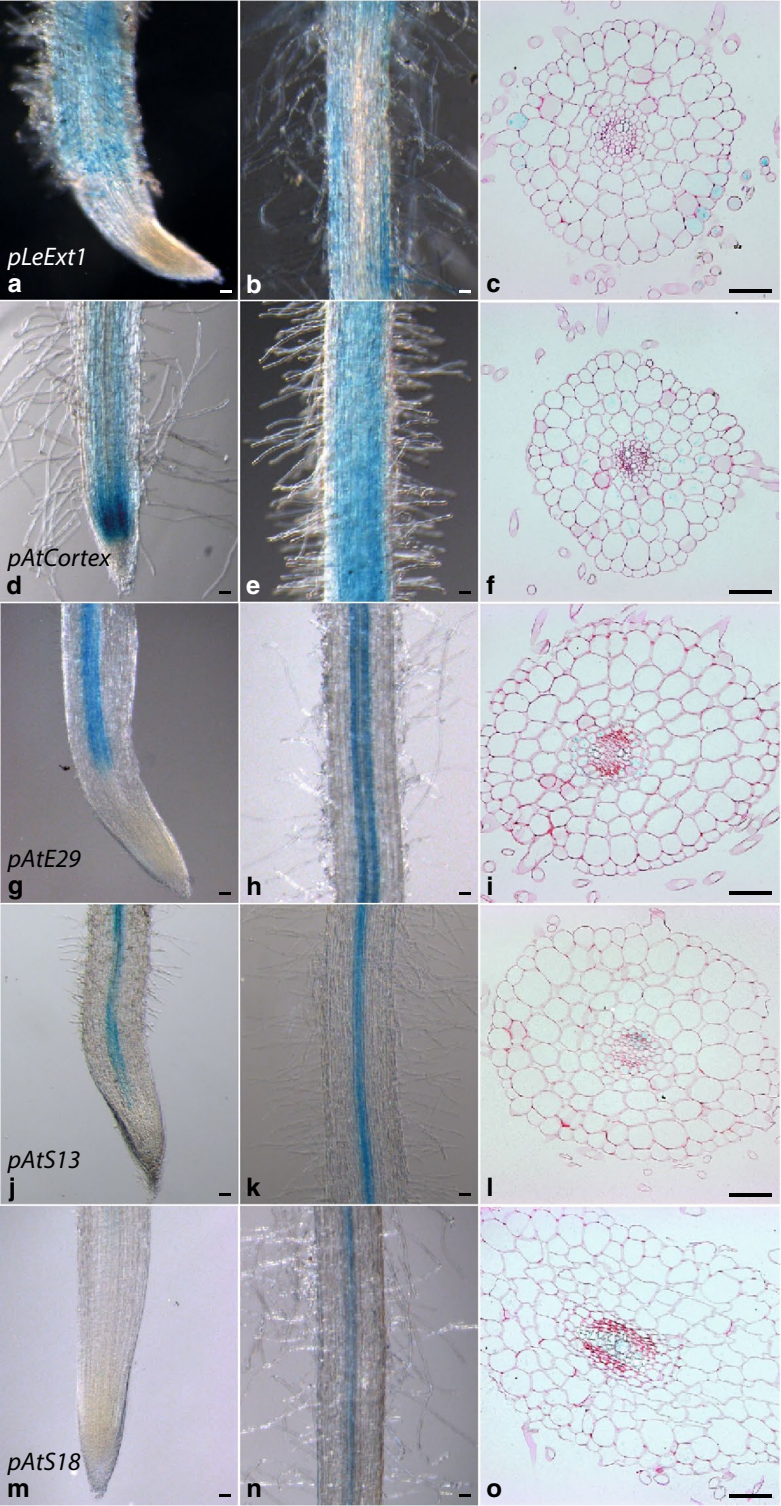
Patterns of cell type enriched promoter activity as indicated by GUS staining are maintained following whole plant regeneration from calli using selected promoter:*GUS* constructs. Transgenes are **a**–**c** *pLeExt1:GUS* (line LIG29-2); **d**–**f** *pAtCortex:GUS* (line CoG11-6); **g**–**i** *pAtE29:GUS* (line 29G4b-5); **j**–**l** *pAtS13:GUS* (line 13G26b-28) and **m**–**o** *pAtS18:GUS* (line XG5A-5). Uninoculated transgenic plants were harvested 3 weeks post germination. Representative GUS stained root tips (**a**, **d**, **g**, **j**, **m** entire root mounts) and responsive zone fragments (**b**, **e**, **h**, **k**, **n** entire root mounts; **c**, **f**, **i**, **l**, **o** cross sections) are shown. Cross sections are 7–8 µm microtome sections of resin (Kulzer Technovit 7100) embedded roots stained with 0.1 % Ruthenium Red.* Scale bars* 50 µm

A selection of promoters including *pLeExt1* (epidermis), *pAtCortex* (cortex), *pAtE29* (endodermis and pericycle), *pAtS13* (phloem and pericycle), and *pAtS18* (xylem) was introduced into transgenic plants to examine whether the processes of tissue culture and plant regeneration from calli influenced the observed expression patterns. Roots of three week-old seedlings homozygous for a single transgene insertion and containing up to two independent insertion sites were analyzed for *GUS* activity. Out of two to four independent lines tested per construct, at least one showed reproducible levels of GUS signal across root systems while maintaining a consistent cell type dependent pattern of GUS staining corresponding to what was previously observed for the respective promoter in transgenic roots (Fig. 2, Table 2, Additional file 1: Fig. S5). The remaining lines showed similar, low or no detectable promoter activity levels. For each construct, we selected one line that showed consistent GUS signal levels for further characterization (Table 2). In line with our observations in transgenic roots on composite plants, lines expressing *pLeExt1:GUS* (epidermis) and *pAtS18:GUS* (xylem) showed patchy GUS signal along roots (Additional file 1: Fig. S5a and e), whereas lines expressing *pAtCortex:GUS*, *pAtE29:GUS* or *pAtA13:GUS* showed mostly continuous GUS signal (Additional file 1: Fig. S5b–d). Activity levels of *pAtCortex:GUS* were consistently more intense in younger root zones including the responsive zone, as compared to mature root sections (Additional file 1: Fig. S5b). Importantly, despite variation in overall activity levels, no lines were observed where the patterns of cell type enrichment deviated from what we observed in composite plants. This suggests that in the tested cases, promoter activity patterns were independent of whether transgenic roots on composite plants or whole plant transformants were analyzed.

**Table 2:** Stable lines expressing selected promoter:*GUS* constructs

Promoter	Line name	Replica	Total plant #	Total GUS+	Total GUS−	%GUS+
*pLeExt1*	LIG29-2	3	18	18	0	100
	LIG31A-19	2	11	11	0	100
	LIG26-7	2	11	9	2	81.82
*pAtCortex*	CoG11-6	3	13	13	0	100
	CoG29-1	2	10	10	0	100
	CoG5b-23	2	10	0	10	0
*pAtE29*	29G4b-5	3	15	15	0	100
	29G7-32	2	15	15	0	100
*pAtS13*	13G26b-28	3	14	14	0	100
	13G3b-100	2	11	11	0	100
*pAtS18*	XG5A-5	3	19	19	0	100
	XG2C-7	2	10	0	10	0
*pCaMV35S*	SG15A-2	2	11	11	0	100
	SG19b-5	2	10	0	10	0
Control vector	NEG15B-5	3	18	0	18	0

Lines selected for onward work based on stable expression patterns are underlined

GUS+, number of transgenic roots where a GUS signal was detected. GUS−, number of transgenic roots with no detectable blue staining. %GUS+, percentage of GUS+ roots

#### Nodule expression patterns

**Fig. 3:**
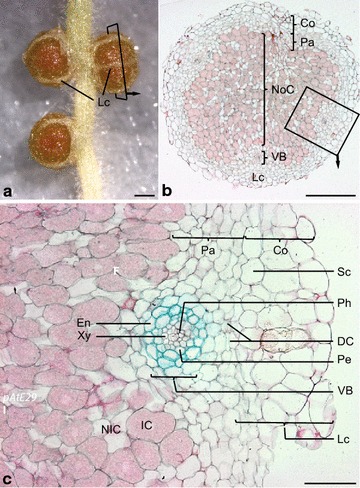
Anatomy of *L.* *japonicus* nodules. **a** Mature nodules on a wild type plant, ecotype Gifu. Lenticels (Lc) allowing for air exchange are apparent as white stripes on the nodule surface. The *rectangle* indicates the plane of a cross section as shown in (**b**). **b** Cross section through a wild type nodule. Cells of the nodule centre [57] infected with *M.* *loti* bacteria appear pink due to Ruthenium Red staining and co-exist with non-infected cells. The *rectangle* indicates an excerpt as shown in **c**. **c** Close-up of a nodule vascular bundle and lenticel and associated inner and outer nodule cell types. The excerpt is a reproduction of Fig. 4l and shows a section of a GUS-stained nodule expressing *pAtS13:GUS* with activity in the vascular endodermis (En) and pericycle (Pe). *DC* Dividing cells, *IC* infected cell, *Pa* nodule parenchyma, *Lc* lenticel, *NIC* interstitial non-infected cell of the nodule centre, *NoC* nodule centre, *VB* vascular bundle, *Co* outer nodule cortex, *Ph* phloem cells, *Sc* sclereid cell layer surrounding the nodule, *En* vascular endodermis, *Pe* vascular pericycle, *Xy* xylem cells. Nomenclature follows Guinel [58], as well as the suggestion of van de Wiel et al. [59] to refer to the inner nodule cortex as nodule parenchyma. Cross sections are 7–8 µm microtome sections of resin (Kulzer Technovit 7100) embedded roots stained with 0.1 % Ruthenium Red.* Scale bars* **a** 1 mm; **b** 50 µm; **c** 20 µm

To evaluate the suitability of the promoters for directed expression studies at later stages of nodulation symbiosis in *L.* *japonicus*, we investigated the activity patterns of *pLeExt1:GUS*, *pAtCortex:GUS*, *pAtE29:GUS*, *pAtS13:GUS*, and *pAtS18:GUS* in nodule cell types (Fig. 3a–c) using selected transgenic lines (Table 3). Plants were harvested at 14 days post inoculation, where the oldest nodules are mature and nitrogen fixing, but senescence has not yet set in. At this timepoint, mature and younger, immature nodules co-exist, allowing for their simultaneous analysis.

**Table 3:** Promoter:*GUS* activity in 14 dpi nodules

Promoter	Line name	Total plant #	Mature nodules (GUS+/total)	Immature nodules (GUS+/total)
*pLeExt1*	LIG29-2	33	0/134	0/63
*pAtCortex*	CoG11-6	16	62/62	48/48
*pAtE29*	29G4b-5	19	69/69	24^a^/25
*pAtS13*	13G26b-28	25	131/131	12^b^/30
*pAtS18*	XG5A-5	32	^c^/127	^c^/57
Control vector	NEG15B-5	34	0/134	0/49

^a^Staining in immature nodules of this line followed the extent of the vascular bundles. Where these had not developed staining was limited to the nodule bases

^b^Immature nodules were unstained in early stages but showed increasing degrees of GUS staining towards reaching maturity

^c^Staining was difficult to trace reliably based on stereolupe inspection, so no number is provided here

GUS+, number of nodules where a GUS signal was detected. Nodules were harvested at 14 days post inoculation with *Mesorhizobium loti* (dpi)

**Fig. 4:**
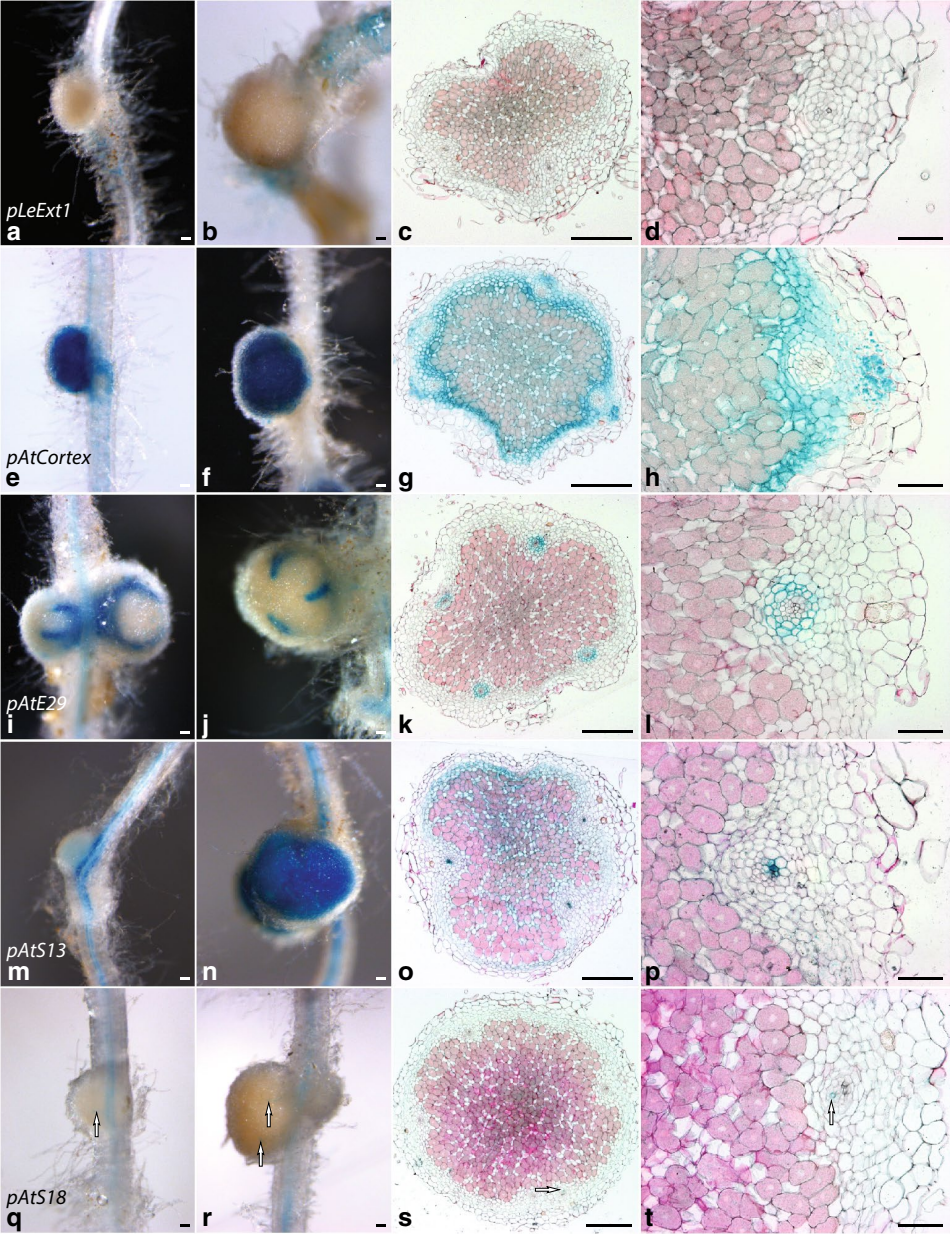
Promoter activity as indicated by GUS staining in immature and mature nodules on transgenic plants expressing selected promoter:*GUS* constructs. Transgenes are **a**–**d** *pLeExt1:GUS* (line LIG29-2); **e**–**h** *pAtCortex:GUS* (line CoG11-6); **i**–**l** *pAtE29:GUS* (line 29G4b-5); **m**–**p** *pAtS13:GUS* (line 13G26b-28) and **q**–**t** *pAtS18:GUS* (line XG5A-5). Plants were inoculated with *M.* *loti* at 1 week of age and harvested at 2 weeks post germination. Representative GUS stained immature (**a**, **e**, **i**, **m**, **q**) and mature (**b**–**d**, **f**–**h**, **j**–**l**, **n**–**p**, **r**–**t**) nodules. Entire mounts (**a**–**b**, **e**–**f**, **i**–**j**, **m**–**n**, **q**–**r**) or cross sections (**c**–**d**, **g**–**h**, **k**–**l**, **o**–**p**, **s**–**t**) are shown. Cross sections are 7–8 µm microtome sections of resin (Kulzer Technovit 7100) embedded roots stained with 0.1 % Ruthenium Red.* Scale bars* **d**, **h**, **l**, **p**, **t** 20 µm, else 50 µm

*pLeExt1:GUS* expressing plants showed no detectable GUS signal in nodule cells (Table 3; Fig. 4a–d). Although root epidermal cells in direct vicinity of nodules regularly showed GUS signal, signal was never detected on root hairs directly associated with nodules, independent of whether cells of the original root epidermis still persisted on emerging primordia or had been replaced by secondary external tissue (Fig. 4a, b). Compared to either non-inoculated roots or inoculated roots that had not developed nodules at the time of harvest, nodulated roots showed a reduced level of epidermal staining (Additional file 1: Fig. S6a).

Nodules on roots expressing *pAtCortex:GUS* showed intense GUS signal at all observed developmental stages (Table 3; Fig. 4e–h). The outer nodule cortex was free of GUS signal, a noteworthy exception being central lenticel cells flanking the vascular bundels (Fig. 4g, h). Phloem, xylem and vascular pericycle were usually unstained (Fig. 4g, h). In contrast, the vascular endodermis as the innermost cortical layer framing the vascular bundles (Fig. 3) showed weak staining in all tested nodules. This is consistent with the observed activity pattern of this promoter in the root responsive zone (Fig. 2f). GUS activity was most pronounced in the nodule parenchyma, a ring of tightly packed cortical cells surrounding the nodule centre (Fig. 3), but both infected and interstitial non-infected cells of the central nodule tissue also showed consistent staining albeit at a weaker level (Fig. 4g, h). In line with the observation that *pAtCortex:GUS* activity was primarily seen in young root sections including the root tip and differentiation zone, but to a lesser extent in mature root sections (Additional file 1: S5b), root sections carrying mature nodules showed low levels or no GUS staining in the root cortex (Additional file 1: Fig. S6b).

*pAtE29:GUS* showed consistent activity in the vascular endodermis surrounding nodule vascular bundles (Figs. 3c, 4i–l). In line with our observations in roots (Fig. 2g–i; Table 1), staining was also present in the vascular pericycle, but at a lower intensity (Fig. 4l). No GUS staining was detected in other cell types of *pAtE29:GUS* expressing nodules (Fig. 4k, l). Similar to *pAtCortex:GUS*, activity of *pAtE29:GUS* in roots was most prominent in younger root sections, but occasionally co-existed with the presence of mature nodules (Additional file 1: Fig. S6b–c).

*pAtS13:GUS* was active throughout the nodule parenchyma and in uninfected central cells of *pAtS13:GUS* expressing nodules (Fig. 4m–p). Activity was already apparent in primordia but at low levels (Fig. 4m), and mature nodules showed intense GUS staining (Fig. 4n). Cross sections revealed that despite this cortical activity, *pAtS13:GUS* was clearly most strongly expressed in phloem cells of the nodule vascular bundles (Fig. 4o, p). Root phloem expression of this promoter was detectable throughout nodulated roots (Additional file 1: Fig. S6i).

Nodules of roots expressing *pAtS18:GUS* showed GUS staining restricted to xylem elements of nodule vascular bundles (Fig. 4q–t). This activity was traceable also in young nodules once vascular bundles began to differentiate (Fig. 4q). Cross sections revealed that only a subset of xylem elements were affected, but reliable quantification was impaired by the difficulty of visually tracing the highly specific GUS signal through lenticel cell layers from the outside, as well as the fact that cross sections only grant a spatially limited insight. *pAtS18:GUS* activity in root xylem elements resembled that observed in uninoculated roots (Additional file 1: Fig. S6e).

#### Degree of cell type enrichment

Cell type enrichment was especially evident upon analysis of thicker preparations from hand or vibratome sectioned roots (Additional file 1: Fig. S3). Such sections were used for analyzing larger numbers of roots but were less suitable for photography using a single focal plane. Three to five representative samples from two to three independent replicates were therefore embedded for the preparation of semithin sections and used for visual documentation. This revealed that with the exception of the weakly active *pAtS18* (proto- and metaxylem), none of the tested promoters seemed entirely limited in activity to the cell type of primary expression. While there usually was a pronounced enrichment of activity in one particular cell type, this was often accompanied by lower level expression activity in one or more other cell types (Figs. 1, 2, 4, Additional file 1: Fig. S1, Table 1). Some of the presented promoters, such as *pAtRHS14* (epidermis and cortex), *pAtE29* (endodermis and pericycle) or *pAtS32* (phloem and pericycle) will thus be of interest primarily in approaches targeting combinations of adjacent cell types.

Unspecific processing of the GUS substrate X-Gluc (5-bromo-4-chloro-3-indolyl glucuronide) by endogenous enzymes is an unlikely cause of background staining, as control roots transformed with a vector lacking the promoter:*GUS* expression cassette showed no blue signal (Additional file 1: Fig. S1.4g–l; Tables 2,3, Additional file 2: Table S1).

Signal leakage from the cells of *GUS* expression to surrounding cells may have contributed to its presence in cells neighboring the cell type of primary activity. Fixing the transgenic roots in advance of applying X-Gluc, which to our experience significantly increased staining specificity while decreasing sensitivity, reduced the amount of GUS signal below the visual detection limit in all cases except the strong *pCaMV35S* positive control. This reflects that all tested promoters were moderate in overall expression intensity, an important aspect that will benefit applications aiming to perform directed expression of target genes in selected cell types and testing for biological effects while avoiding artifacts related to overexpression.

For a subset of promoters including *pLeExt1*, *pAtS5*, *pAtS13*, *pAtS32* and *pAtE30* from the list of those showing detectable activity using GUS fusions, we have prepared additional marker fusion constructs using the triple yellow fluorescent protein (tYFP) locating to the cell cytoplasm. However, only the strong *pCaMV35S* promoter gave a reproducible, screenable signal above auto-fluorescence of *L.japonicus* roots. Based on previous evidence indicating a higher sensitivity of the GUS reporter system compared to cytoplasm localized green fluorescent protein [46] we thus turned to promoter:*GUS* fusions for our further analyses.

In contrast, using transcriptional and/or translational fusions to GFP localized to the endoplasmatic reticulum, Lee et al. could detect expression of 79 % of the 61 cell type enriched promoters selected based on microarray expression data obtained from root cell type specific cell populations [6, 10] in the native Arabidopsis. Where the expression pattern detected with fluorescent markers resembled that established via transcriptome analysis, fluorescence was seen only in the most strongly represented cell types [6, 10]. Similarly, our results suggest that while the tested promoters may indeed show broader expression ranges in *L.japonicus* compared to the endogenous Arabidopsis, higher detection sensitivity using the GUS reporter system may reveal low levels of expression present in other than the dominant cell type(s). In line with this, for the xylem enriched promoter *pAtS18,* where activity was exclusively seen in xylem cells in *L.* *japonicus* roots based on GUS signal analysis, ca. 96 % of the transcripts detected in the microarray [10] stemmed from the xylem-fraction.

## Conclusions and perspectives

We present a promoter toolbox for cell type enriched expression analysis in roots and nodules of the model legume *L. japonicus*, which we expect to significantly contribute to our understanding of nodulation symbiosis as well as root development in legumes. Beyond assisting in the analysis of individual genes’ function in *L. japonicus* roots and nodules, the promoters presented in this study can provide a stepping-stone for global translatome analysis of *L.japonicus* roots undergoing initial stages of symbiosis. Such analyses will significantly improve the resolution of our current understanding of root symbiosis from the organ- to the cell type level.

## Methods

### Biological material

Cloning works made use of *E. coli* strains TOP10 or DB3.1. *Agrobacterium tumefaciens* AGL1 [47] was used for whole plant transformation and *Agrobacterium rhizogenes* AR1193 [48] for transgenic root generation. *Mesorhizobium loti* MAFF 303099 expressing *DsRED* [49] for *L.japonicus* infection experiments. Promoter expression analysis was performed in transgenic roots of *L. japonicus* ecotypes MG20 [50] and Gifu B-129 [51].

### Promoter isolation

Promoter candidates were selected based on published expression patterns in plant roots and amplified for sequence cloning and verification from genomic DNA of *A.thaliana* var. Col-0 or from plasmid templates (*pAtSUC2*, *pLeExt1*). A pENTR/D-TOPO (Invitrogen, Life Technologies) based construct containing pE30 [6] was kindly provided by P. Benfey (Duke University, Durham, USA) and used for recombination into expression vectors.

### Expression construct generation

To analyze their expression patterns in *L. japonicus* roots, promoter fragments were inserted before an intron-containing *β*-*glucuronidase* (*GUS*) coding sequence [52] followed by a *CaMV35S* terminator using a Gateway (Invitrogen, Life Technologies)-compatible derivative of a pIV10 [48] integration plasmid (selection: 100 mg/l ampicillin and 100 mg/l spectinomycin). Upon transformation into appropriate *A.rhizogenes* the latter recombines into the transfer-DNA of the agrobacterial root inducing plasmid [48]. Gateway recombination reactions were done following manufacturers’ instructions (Invitrogen, Life Technologies).

Constructs for stable transformation are based on a pGreenII0029 (selection: kanamycin 50 mg/l) binary plasmid equipped with a Gateway (Invitrogen, Life Technologies) destination cassette followed by *GUS* coding and *CaMV35S* terminator sequences as used in the pIV10 construct employed in transgenic root generation.

Fragments for cloning were amplified using Phusion High-Fidelity DNA Polymerase (Fermentas, Life Technologies) following manufacturers’ instructions. All intermediate and final constructs were confirmed by Sanger sequencing. Primers used for promoter fragment amplification and cloning are listed in Additional file 2: Table S2.

### Plant growth and transformation

*Lotus japonicus* seeds were scarified in sulphuric acid for up to 20 min depending on age and subsequently surface sterilized in 0.5–1 % sodium hypochlorite solution for 20 min. Seeds were then imbibed at 4 °C overnight and grown in a 16 h light/8 h dark regime at 21/16 °C, respectively. Transgenic roots were generated as previously described [31]. Composite plants were grown on 12 × 12 cm square cultivation dishes on wedged 0.5 × strength Gamborg B5 medium (Duchefa Biochemistry) supplemented with 1 % Agar Noble (Sigma-Aldrich) for 6 weeks. They were then transferred to magenta growth containers (Sigma-Aldrich) with a 4:1 mix of Leca clay granules (Optiroc) and Vermiculite in 60 ml of 0.25 % Broughton and Dilworth medium [53] supplemented with 1 mM KNO_3_ (B&D). After 10 days, plants were either mock treated with 20 ml ¼ strength B&D medium or inoculated with 20 ml of an *M.loti* suspension in ¼ strength B&D at an optical density of 0.001 at λ = 600 nm.

Whole plant transformation of *L.* *japonicus* Gifu seedlings was done using a modified version of an earlier published protocol [54]. Shoots emerging from calli were transferred to soil substrate for rooting at a length of three to four cm and kept under greenhouse conditions until the seeding stage. Plants were genotyped for transgene presence, and seeds were collected from positively scoring plants for the isolation of lines homozygous for one transgene insertion. Seeds of three to four homozygous lines were collected and germinated as described above, then transferred directly to magenta growth containers. After 1 week of growth, plants were either mock treated with 20 ml ¼ strength B&D medium or inoculated with 20 ml of an *M.* *loti* suspension in ¼ strength B&D at an optical density of 0.001 at λ = 600 nm and harvested 2 weeks after inoculation. Cultivation conditions were as described for composite plants.

### Identification of transgene insertion sites in transgenic plants

To isolate lines containing a minimal number of transgene insertions and to facilitate the identification of integration sites, we have adapted Sequence-Specific Amplification Polymorphism (SSAP) analysis in the *L.japonicus* background [55] for pGreenII0029 transfer-DNA. Lines containing one or two transgene insertion sites were considered for onward analysis.

### GUS staining and fixation

GUS staining was done as described by Vitha et al. [56] with few modifications. Briefly, roots were harvested into ice-cold phosphate buffer (50 mM NaH_2_PO_4_, 50 mM Na_2_HPO_4_, pH 7.0). Phosphate buffer was then exchanged for X-Gluc substrate buffer [0.5 mg/ml X-Gluc dissolved in DMSO, 50 mM phosphate buffer, 1 mM K_4_(Fe(CN)_6_), 1 mM K_3_(Fe(CN)_6_), 0.05 % Triton X-100]. Roots were vacuum infiltrated for 10 min and incubated in staining buffer for 12 h at 37 °C.

For direct inspection or embedding in agarose, roots were fixed by vacuum infiltration for 10 min at room temperature, followed by 30 min incubation at 4 °C in a solution of 4 % glutaraldehyde in 50 mM phosphate buffer, pH 7.

For semithin sections, a representative subset of stained root and nodule samples were fixed by vacuum infiltration in a solution of 4 % paraformaldehyde and 1 % glutaraldehyde in 100 mM phosphate buffer (pH 7.2) for 20 min, incubated at 4 °C over night while shaking, then dehydrated in serial dilutions of ethanol in water (15, 30, 45 and 60 %) for 30 min each and stored in 70 % ethanol. Roots were then embedded in Kulzer Technovit 7100 resin (Emgrid Australia) following the manufacturers’ instructions.

### Microscopic analysis

GUS stained, fixed roots and nodules were viewed and documented directly using a Leica M165FC stereomicroscope and Leica DFC 310 FX camera system. Entire roots were analyzed longitudinally, and root zones responsive to rhizobial infection identified by the presence of immature, developing root hairs (differentiation zone) were cross-sectioned to visualize expression patterns in inner root cell types. For analysis of cross sections, roots or nodules were embedded in 3 % agarose and sectioned at 60–80 γm thickness using a vibratome (Leica VT 1000 S, Leica). Vibratome sections of were used for screening larger numbers of roots, and 3–5 representative samples were used to generate semithin sections. Semithin sections (7–8 γm) of resin embedded samples were prepared using a Leica RM2045 microtome and stained with 0.1 % Ruthenium Red (Sigma-Aldrich) in water. Cross sections were analyzed using an Axioplan 2 microscope (Zeiss), and pictures were taken with an AxioCam color camera from the same supplier.

## Additional files

10.1186/s13007-016-0105-y Activity patterns of promoter candidates showing epidermis enriched (**Figure S1.1**), general cortex- or endodermis enriched (**Figure S1.2**), pericycle and phloem enriched (**Figure S1.3**) or xylem specific (**Figure S1.4**) expression in *L.* *japonicus* roots. **Figure S1.4** **g**-**l** show roots expressing a control vector devoid of a promoter:*GUS* expression cassette. *Agrobacterium rhizogenes* induced transgenic roots were harvested three days after mock treatment (mock) with medium or inoculation with *M.* *loti* bacteria. Representative GUS stained root tips (**a**,**d**,**g**,**j**,**m**,**p** entire root mounts) and responsive zone fragments (**b**,**e**,**h**,**k**,**n**,**q** entire root mounts; **c**,**f**,**i**,**l**,**o**,**r** cross sections) are shown. Cross sections are 7-8 µm microtome sections of resin (Kulzer Technovit 7100) embedded roots stained with 0.1 % Ruthenium Red. *Scale bars* 50 µm. **Figure S2.** Root tip associated GUS signal varied with root hair emergence patterns in *pLeExt1:GUS* expressing roots. Where root hairs developed near the root tip, epidermal cells showed GUS activity in a distinct ring of blue encircling the root tip. GUS stained and fixed tips of *Agrobacterium rhizogenes* induced transgenic roots harvested **a** three days after mock treatment (mock) with medium or **b** inoculation with *M.* *loti* bacteria are shown. *Scale bars* 50 µm. **Figure S3.** Vibratome sections of *pAtCortex:GUS* expressing roots show strong enrichment of GUS signal in cortical cells. **a**–**b** 60-80 µm sections of the responsive zone of *pAtCortex:GUS* expressing *L.* *japonicus* roots. Roots were stained, fixed and embedded in 2.4 % agarose prior to sectioning. *Scale bars* 50 µm. **Figure S4.** *pAtSUC2* activity pattern in transgenic *L. japonicus* roots. **a**–**b** roots shielded from light access show no GUS staining. **c**–**d** chloroplast-containing roots exposed to light show GUS signal in phloem cells. *Arrows* indicate GUS signal in chloroplast containing roots. Representative longitudinal views of GUS stained whole root mounts are shown. **e** cross-section of resin (Kulzer Technovit 7100) embedded root responsive zone stained with 0.1 % Ruthenium Red. Black arrows point to phloem poles, white arrows to pericycle cells showing GUS signal. The respective root had been exposed to light during development. *Scale bars* **a**–**d** 100 µm, **e** 50 µm. **Figure S5.** Promoter activity distribution across developmental zones of representative roots stably expressing promoter:*GUS* constructs. Transgenes are **a** *pLeExt1:GUS* (line LIG29-2); **b** *pAtCortex:GUS* (line CoG11-6); **c** *pAtE29:GUS* (line 29G4b-5); **d** *pAtS13:GUS* (line 13G26b-28) and **e** *pAtS18:GUS* (line XG5A-5). Transgenic plants were uninoculated and harvested as well as GUS stained at three weeks post germination. *Scale bars* 1 mm. **Figure S6.** Promoter activity distribution across developmental zones of representative nodulated roots stably expressing promoter:*GUS* constructs. Transgenes are **a** *pLeExt1:GUS* (line LIG29-2); **b** *pAtCortex:GUS* (line CoG11-6); **c** *pAtE29:GUS* (line 29G4b-5); **d** *pAtS13:GUS* (line 13G26b-28) and **e** *pAtS18:GUS* (line XG5A-5). Transgenic plants were harvested and GUS stained at two weeks after inoculation with *M.* *loti*. *Scale bars* 1 mm.
10.1186/s13007-016-0105-y *L. japonicus* roots showing GUS staining when transformed with the tested promoter:*GUS* constructs. **Table S2.** Oligonucleotide primers used for amplification and cloning of promoter fragments presented in this manuscript.
