# Supplementary figures and images for: Inoculation insensitive promoters for cell type enriched gene expression in legume roots and nodules
Source: Plant Methods. 2016 Jan 22;12:4. doi: 10.1186/s13007-016-0105-y (PMC4724153; doi:10.1186/s13007-016-0105-y)

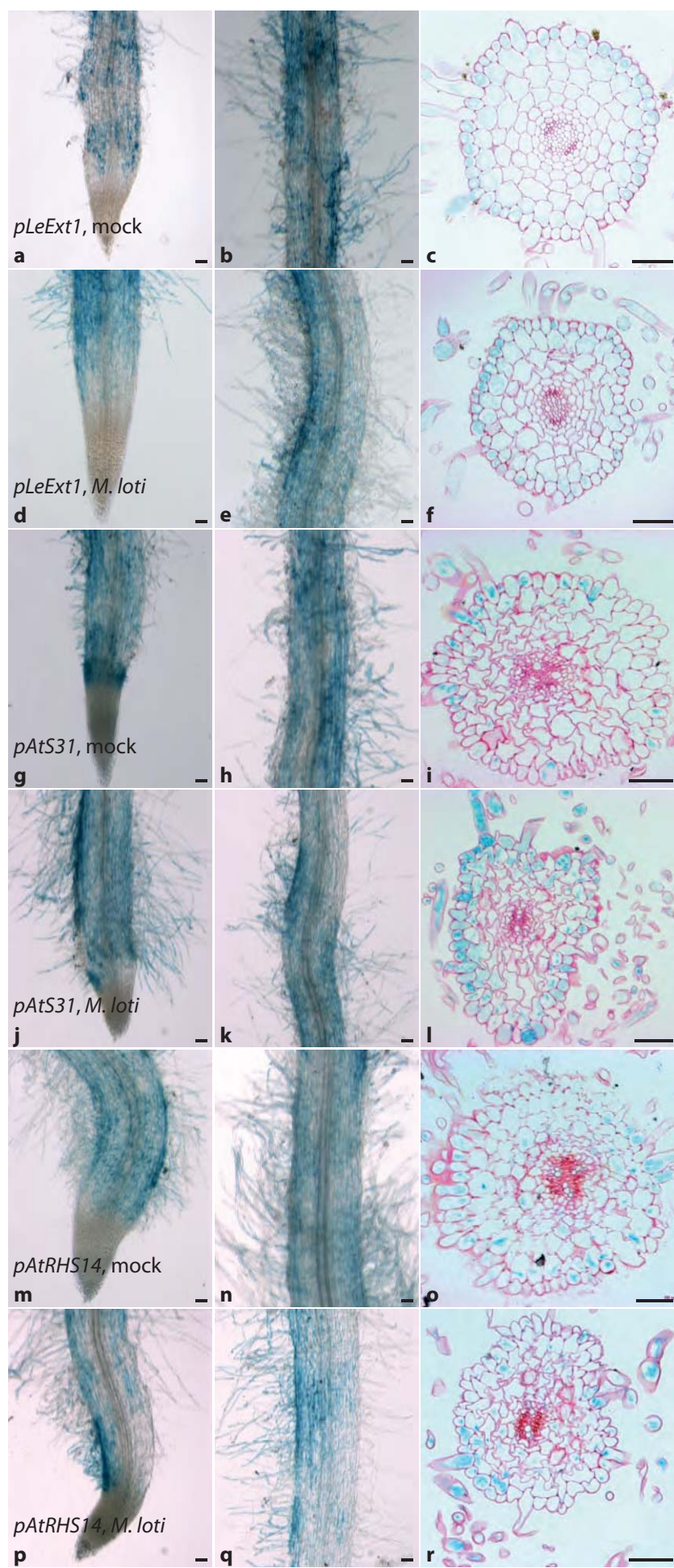

Figure S1.1

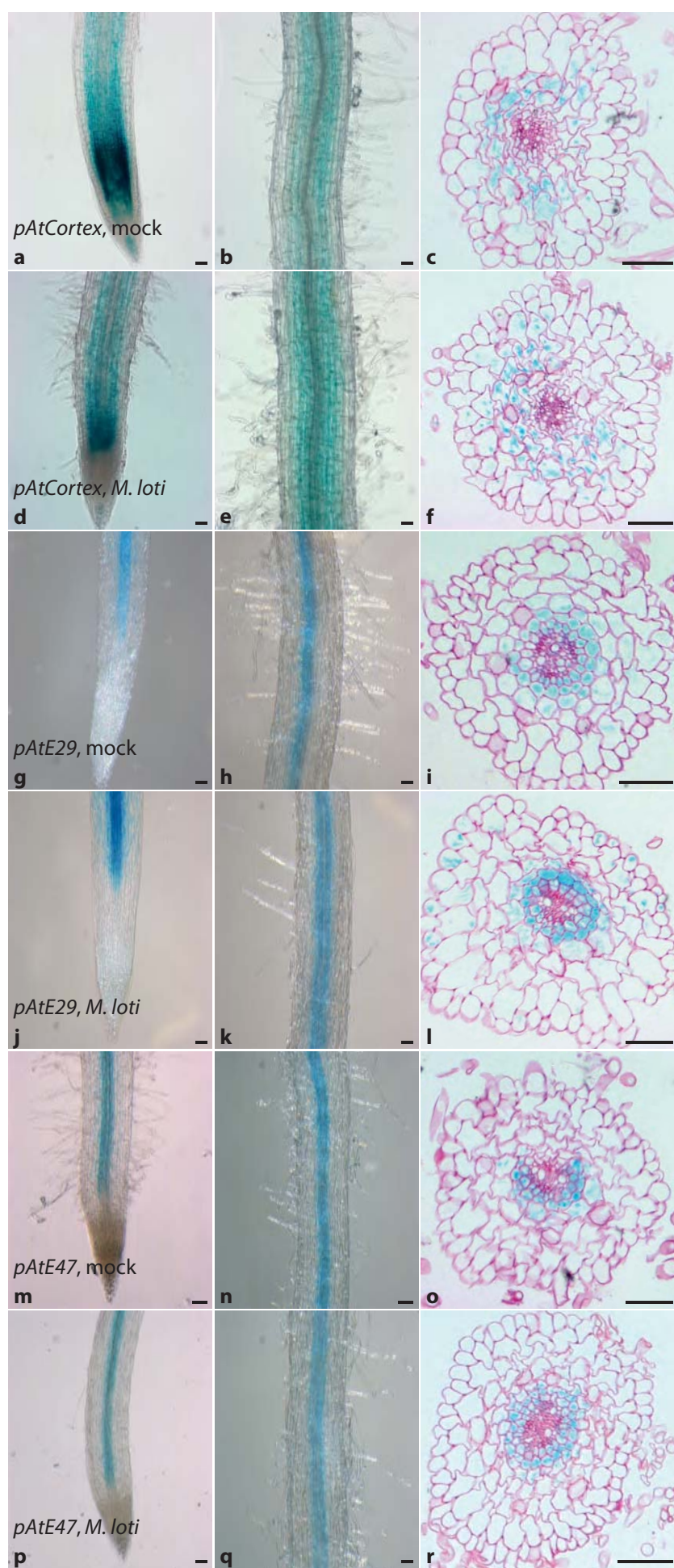

Figure S1.2

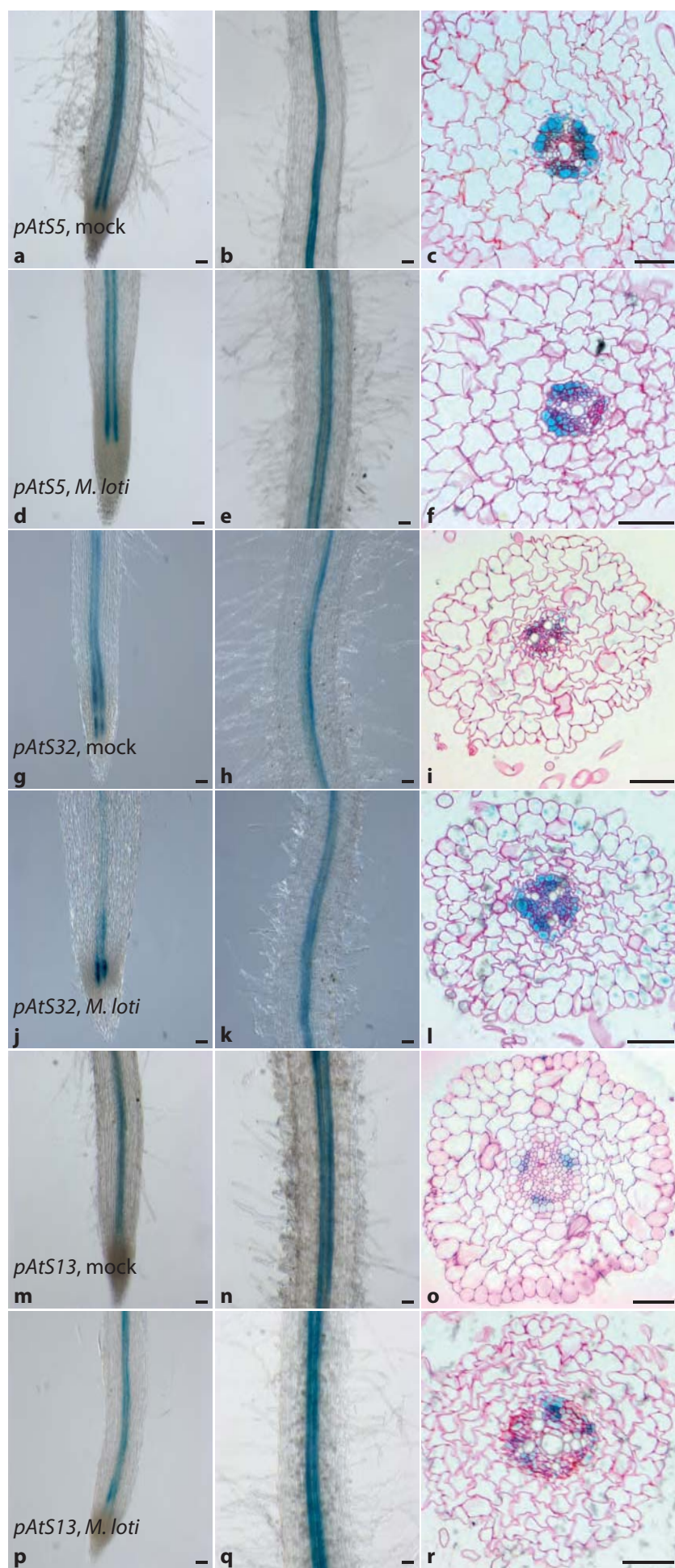

Figure S1.3

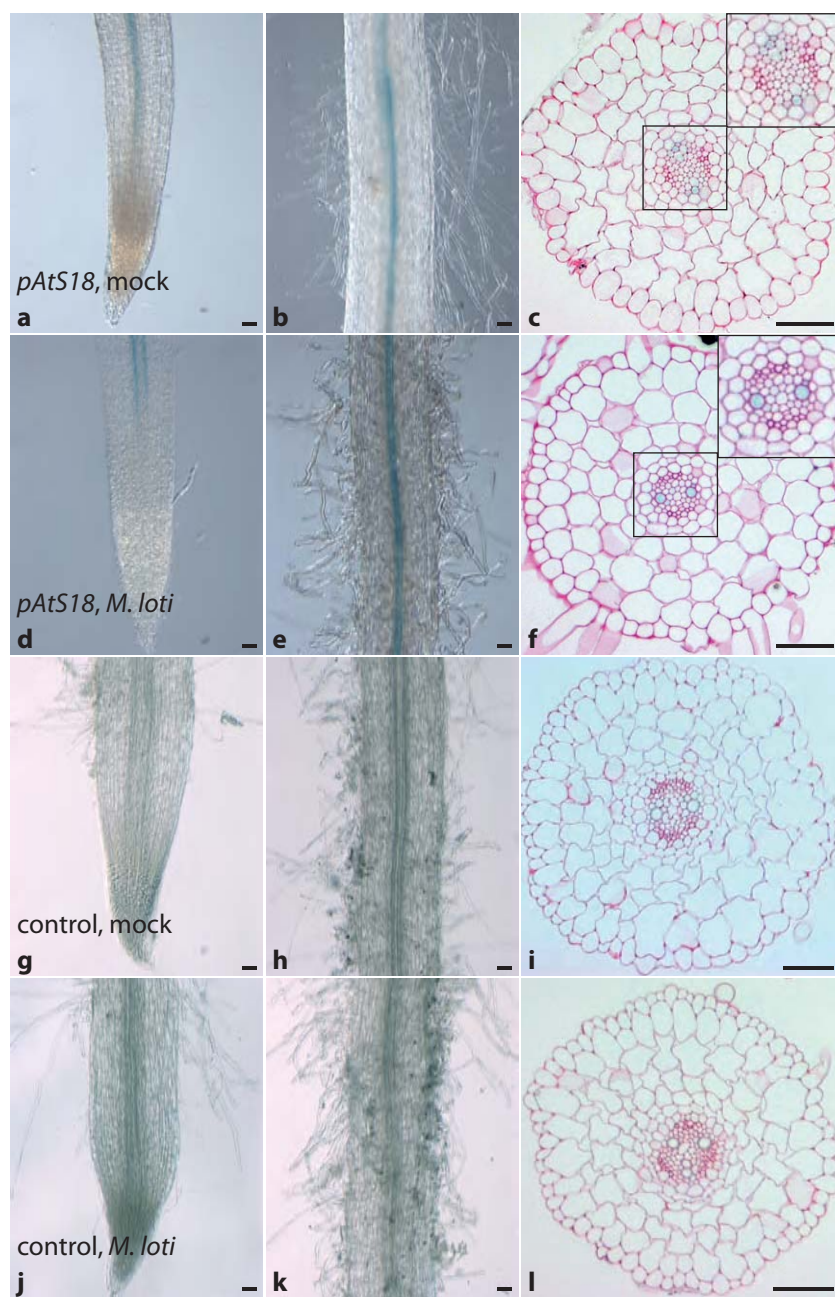

Figure S1.4

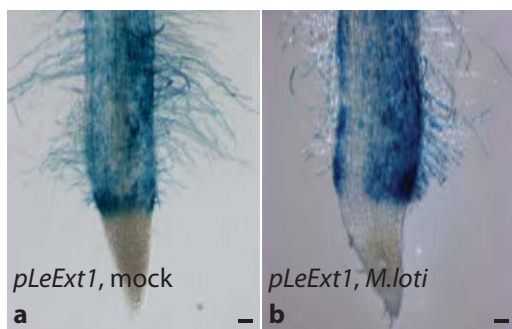

Figure S2

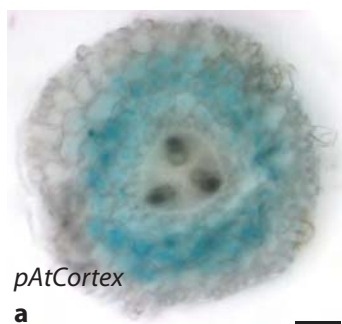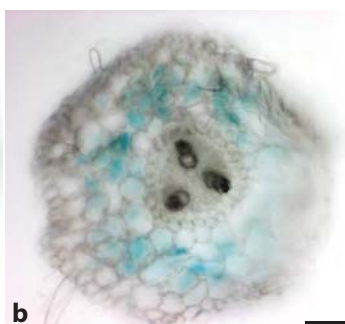

Figure S3

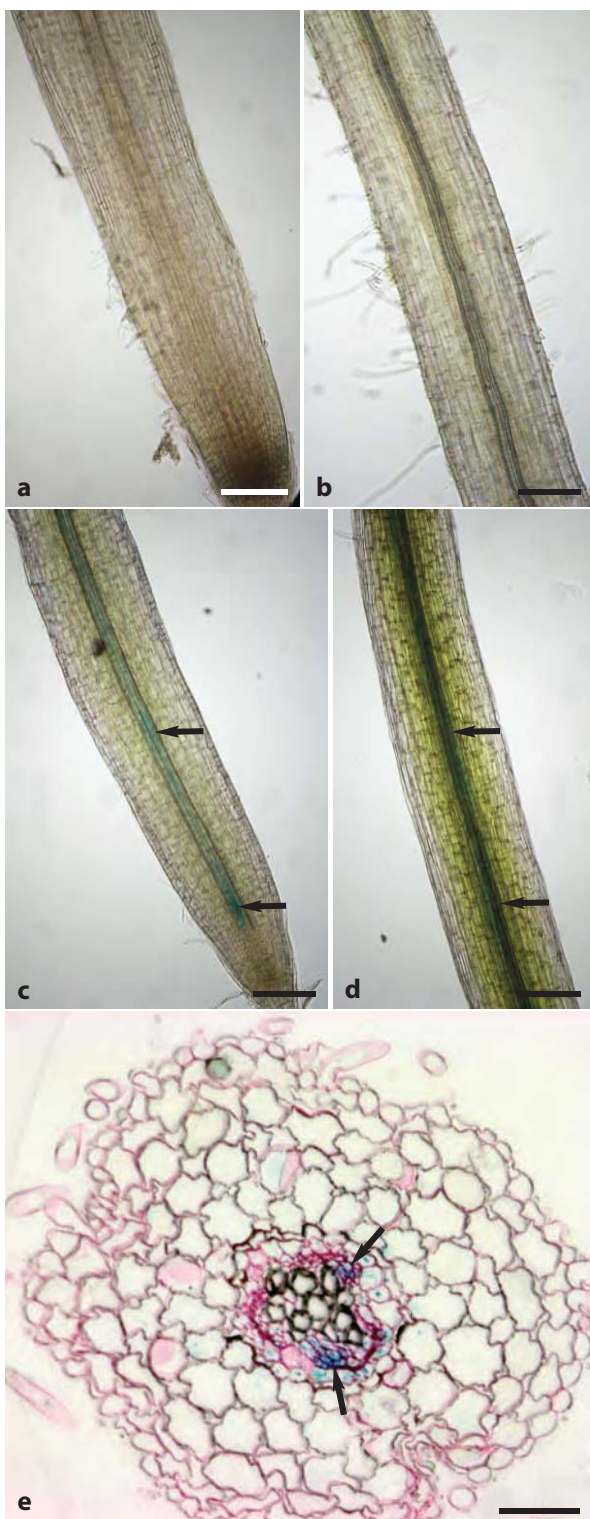

Figure S4

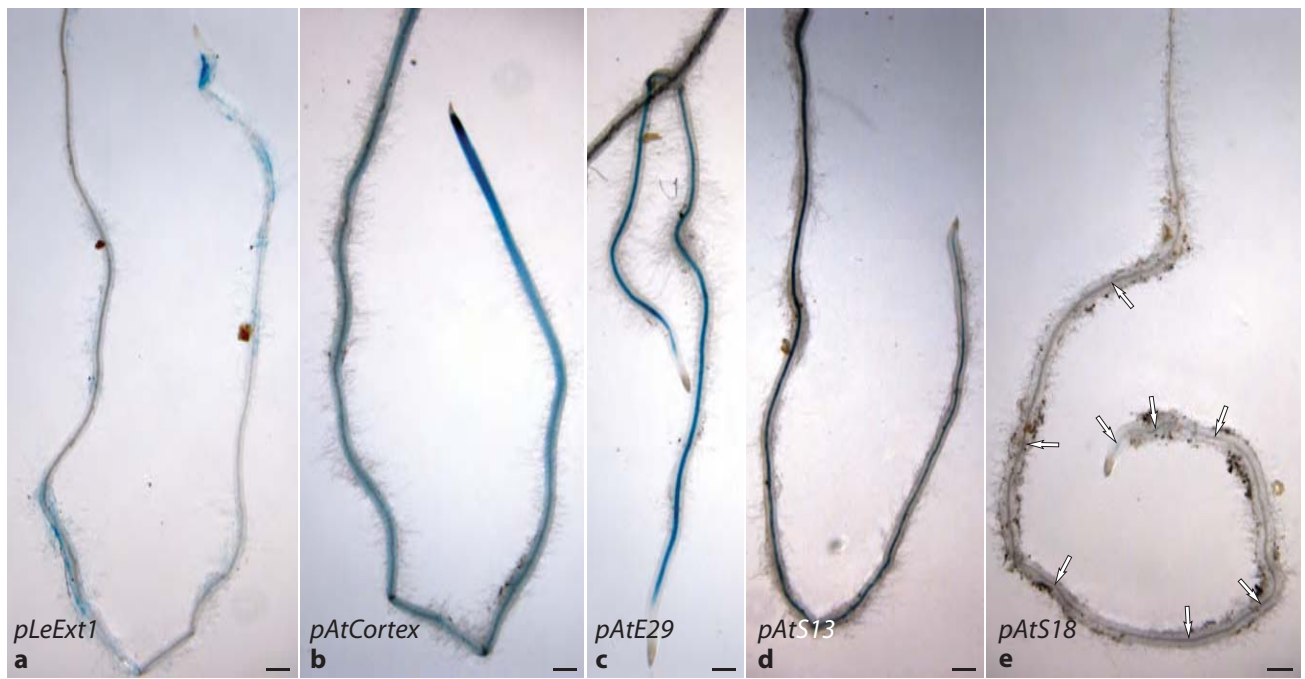

Figure S5

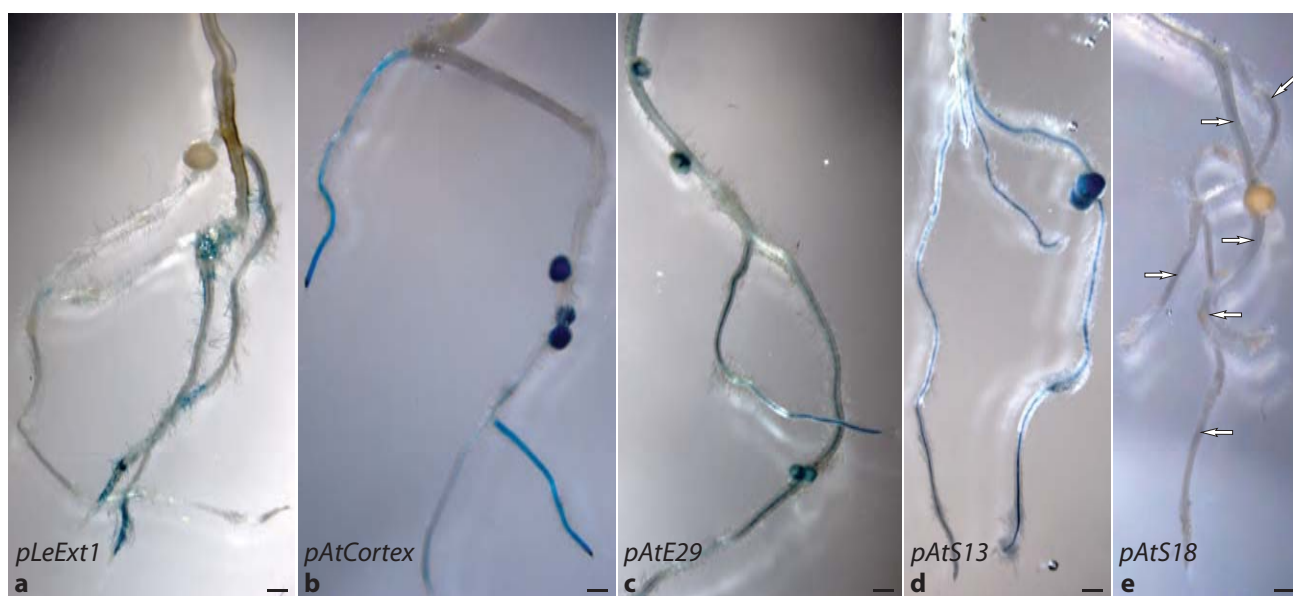

Figure S6

Supplement: Supplementary file 1 — 10.1186/s13007-016-0105-y Activity patterns of promoter candidates showing epidermis enriched (Figure S1.1), general cortex- or endodermis enriched (Figure S1.2), pericycle and phloem enriched (Figure S1.3) or xylem specific (Figure S1.4) expression in L. japonicus roots. Figure S1.4 g-l show roots expressing a control vector devoid of a promoter:GUS expression cassette. Agrobacterium rhizogenes induced transgenic roots were harvested three days after mock treatment (mock) with medium or inoculation with M. loti bacteria. Representative GUS stained root tips (a,d,g,j,m,p entire root mounts) and responsive zone fragments (b,e,h,k,n,q entire root mounts; c,f,i,l,o,r cross sections) are shown. Cross sections are 7-8 µm microtome sections of resin (Kulzer Technovit 7100) embedded roots stained with 0.1 % Ruthenium Red. Scale bars 50 µm. Figure S2. Root tip associated GUS signal varied with root hair emergence patterns in pLeExt1:GUS expressing roots. Where root hairs developed near the root tip, epidermal cells showed GUS activity in a distinct ring of blue encircling the root tip. GUS stained and fixed tips of Agrobacterium rhizogenes induced transgenic roots harvested a three days after mock treatment (mock) with medium or b inoculation with M. loti bacteria are shown. Scale bars 50 µm. Figure S3. Vibratome sections of pAtCortex:GUS expressing roots show strong enrichment of GUS signal in cortical cells. a–b 60-80 µm sections of the responsive zone of pAtCortex:GUS expressing L. japonicus roots. Roots were stained, fixed and embedded in 2.4 % agarose prior to sectioning. Scale bars 50 µm. Figure S4. pAtSUC2 activity pattern in transgenic L. japonicus roots. a–b roots shielded from light access show no GUS staining. c–d chloroplast-containing roots exposed to light show GUS signal in phloem cells. Arrows indicate GUS signal in chloroplast containing roots. Representative longitudinal views of GUS stained whole root mounts are shown. e cross-section of resin (Kulzer Tec [file 13007_2016_105_MOESM1_ESM.pdf]
